# Direct Observation of the Behaviour of Females with Rett Syndrome

**DOI:** 10.1007/s10882-016-9478-0

**Published:** 2016-03-04

**Authors:** Rina Cianfaglione, Andrea Meek, Angus Clarke, Michael Kerr, Richard P. Hastings, David Felce

**Affiliations:** Welsh Centre for Learning Disabilities, Institute of Psychological Medicine and Clinical Neurosciences, Cardiff University, 2nd floor Hadyn Ellis Building, Maindy Road, Cardiff, Wales CF24 4HQ UK; Institute of Cancer & Genetics, Institute of Medical Genetics Building, Cardiff University, Heath Park, Cardiff, CF14 4XN UK; Centre for Educational Development Appraisal and Research, University of Warwick, Coventry, CV4 7AL UK

**Keywords:** Intellectual disabilities, Rett syndrome, observation of behaviour, activity, self-injury

## Abstract

The aim was to observe the behaviour of a sample of females with RTT and explore how it was organized in relation to environmental events. Ten participants, all with a less severe form of classic (*n* = 9) or atypical (*n* = 1) Rett syndrome (RTT), were filmed at home and at school or day centre. Analysis used real-time data capture software. Observational categories distinguished engagement in social and non-social pursuits, hand stereotypies, self-injury and the receipt of attention from a parent, teacher or carer. Associations between participant behaviour and intake variables and receipt of attention were explored. Concurrent and lagged conditional probabilities between behavioural categories and receipt of attention were calculated. Receipt of adult attention was high. Engagement in activity using the hands was associated with a less severe condition and greater developmental age. Engagement in activity, whether using the hands or not, and social engagement were positively associated with receipt of support. The extent of hand stereotypies varied greatly across participants but was independent of environmental events. Six participants self-injured. There was some evidence that self-injury was related to adult attention. Participants appeared to experience a carer and attention rich environment and their levels of engagement seemed high as a result. As in the more general literature, engagement in activity was related to personal development and to social support. Self-injury contrasted with hand stereotypies in having possible environmental function.

## Introduction

Rett syndrome (RTT) affects almost exclusively females, with an incidence of up to one in every 10,000 live female births. Its cause is most often a mutation in the methyl-CpG binding protein-2 (*MECP2*) gene, located on the X chromosome at *Xq28* (Amir et al. 1999). It is characterised by a period of regression in early childhood, accompanied by loss of purposeful hand skills and language and the development of stereotypic hand movements. This is followed by stabilization and usually ongoing profound intellectual disability. The presence of certain behavioural features in the main or supportive diagnostic criteria (Neul et al. 2010) suggests that RTT syndrome has a definable behavioural phenotype (Cianfaglione et al. 2015a; Mount et al. 2001, 2003). Table 1 lists behaviours, or behavioural signs of possible autonomic disorder such as hyperventilation or breath holding, that are mentioned as occurring either frequently or fairly frequently in six surveys of RTT. Hand stereotypies appear to be pervasive when assessed. Teeth grinding, sleeping difficulties and night-time laughing, screaming, anxiety or inappropriate fear, problems in mood regulation, breathing abnormalities and self-injury may also be expected in the majority or substantial minority.

**Table 1:** Behavioural commonalities between surveys of RTT syndrome

Behavioural characteristic	Percentage of sample with characteristic					
	Coleman et al. (1988) (*N* = 63)	Sansom et al. (1993) (*N* = 107)	Mount et al. (2001) (*N* = 38)	Cass et al. (2003) (*N* = 87)	Halbach et al. (2008) (*N* = 53)	Cianfaglione et al. (2015a) (*N* = 91)
Hand stereotypies	100	-	100	97	-	99
Teeth grinding	95	-	37	-	-	58
Screaming	84	48 (night)	-	-	39 (night)	44
Night unrest/laughing	83	84	21	-	77	64
Anxiety/Inappropriate fear	75	75	-	-	68	73
Low mood/mood changes	-	70	-	-	66	77
Hyperventilation	63	32	84	60	39	63
Breath hold	57	-	37	41	73	77
Self-injury	49	48	-	73	-	28

- Behavioural characteristic not included in the survey

The extent to which environmental variables may account for behaviour in females with RTT remains largely unexplored as few studies have involved systematic observation of their behaviour in relation to their surroundings. A number of single case studies have explored the functionality of self-injury and hand stereotypies. Oliver et al. (1993) found that the function of the self-injury of a child with RTT was to terminate social contact, whereas Iwata et al. (1986) concluded that the hand biting of two individuals was independent of environmental circumstances and appeared to be self-stimulatory. Wehmeyer et al. (1993); Roane et al. (2001) and Wales et al. (2004) conducted functional assessments of hand stereotypies. All but one analysis suggested that occurrence was at a high rate and unaffected by environmental conditions.

The aims of this study were to add to and broaden the focus of observational research on RTT by exploring how the behaviour of a sample of females with RTT with a confirmed *MECP2* mutation was organized in relation to environmental events. In particular, as functional hand use is lost in RTT, one objective was to assess the extent of constructive activity that individuals engaged in and the extent to which such engagement relied on social support. A second objective was to examine how seemingly characteristic or socially significant behaviours such as hand stereotypies or self-injury occurred within the sequence of behaviour and whether they had any discernable environmental antecedents or consequences.

## Methods

### Sampling and Participant Characteristics

Before commencing the study, ethical approval was granted by the NHS Research Ethics Committee for Wales (Application number: 09/MRE09/50). In a prior stage, a national sample of 91 females with RTT had been recruited by contacting families known to the on-going British Isle Rett Syndrome Survey database (Cianfaglione et al. 2015b). The ages of sample members ranged from 4 to 47 years with a mean of 20.5 years: 43 were children and 48 adults. Seventy-one (78.0 %) were known to be *MECP2* positive. Invitation letters to participate in the direct observational study were sent to 25 of these 71 families and 16 agreed to take part. However, due to the practicalities of travel and constraints on research time, only 11 were visited. Nine had diagnoses of classic RTT, one of atypical RTT and one of *MECP2* related disorder. This last person has subsequently been excluded but we have chosen to include the individual with atypical RTT, although we have taken care to discuss her results separately from those of the other nine participants with classic RTT. She was categorised as atypical as a regression in language was not noted because she had no babble and did not speak.

The characteristics of the 10 participants are set out in Table 2. Five were children, one an adolescent and four were adults. Their median age was 12.5 years (range 5–32 years). Median developmental age, as measured by the Vineland Adaptive Behavior Scale – Survey Form (Sparrow et al. 1984 - see below), was 11.0 months (range 8–15 months). One person could walk unsupported. The ability to walk was impaired in seven of the participants and never acquired in the remaining two. Hand use was reduced in four and lost in six. Speech was lost in eight and never acquired in one. Post-stabilisation, the one participant (P1) with atypical RTT had gained a few words. All were reported to have hand stereotypies. All had a mild/less severe clinical phenotype as assessed by the Simplified Severity Score (Smeets et al. 2009 - see below).

**Table 2:** Participant characteristics

Partic- ipant	Chronological age (years)	Developmental age (months)*	Diagnosis	*MECP2* mutation	Mobility	Severity Score**
Children						
P1	5 yrs	13 months	Atypical RTT	c.116delGA	Never Acquired	9
P2	5 yrs	8 months	Classic RTT	R294X	Impaired	6
P3	8 yrs	Not Available	Classic RTT	R255X	Impaired	8
P4	10 yrs	11 months	Classic RTT	P152R	Impaired	5
P5	11 yrs	11 months	Classic RTT	del.exon 4–3	Never Acquired	8
P6	14 yrs	10 months	Classic RTT	P101L	Impaired	9
Adults						
P7	21 yrs	11 months	Classic RTT	R306C	Impaired	4
P8	23 yrs	11 months	Classic RTT	R294X	Impaired	6
P9	28 yrs	15 months	Classic RTT	R306C	Walks unsupported	4
P10	32 yrs	13 months	Classic RTT	R306H	Impaired	6

*As assessed by using the Vineland Adaptive Behavior Scales Survey Form

**The maximum score of 18 indicates the most severe phenotype

### Measurement

Information was collected on date of birth, diagnosis, genetic causation, early development and current status, the latter by completion of the Simplified Severity Score (Smeets et al. 2009) and the Vineland Adaptive Behavior Scale – Survey Form (Sparrow et al. 1984). The Simplified Severity Score addresses six features of RTT: sitting, walking, hand use, speech, epilepsy and spine deformation. Each domain is scored from 0 to 3. The total score, which has a maximum of 18, evaluates the overall severity of the syndrome. Scores of 9 or less are considered mild or less severe. The Vineland Adaptive Behavior Scale Survey Form contains 297 items, which assess adaptive behaviour in people with and without intellectual disabilities. Good internal consistency and inter rater reliability coefficients have been reported (Sparrow et al. 1984).

For the behavioural observation, categories were defined to cover the behaviour of the person with RTT, the social contact received from a parent or carer and the proximity of another person as an environmental condition. Behavioural categories and their definitions are set out in Table 3. In brief, the Engaged Activity category included any self-help, domestic activity/work, leisure/play or educational activity and included simple early years actions that may be appropriate for the person’s developmental level, such as mouthing an object or manipulating a rattle or sensory toy. Engaged activity involving use of the hands was distinguished from that which did not involve the hands (e.g., looking at television or listening to a story). Social Engagement included all behaviours orientated towards another person to obtain and/or maintain interaction, such as vocalizing towards another person, maintaining eye contact with a person, reaching out towards a person or orienting to a person in response to physical or vocal contact. Stereotypic behaviour, self-injury, aggression, breathing abnormalities and Rett episodes (a non-epileptic behaviour often misidentified as a possible seizure in which the eye gaze is not fixed, the person appears to be holding their breath, with absence of hand movements and motor activities) were defined, as was mood. Parent/carer interaction was defined as giving Support to assist the participant to conduct an activity or Help (i.e., attending to the participant to help them feed, drink, dress etc. but in a way that did not assist the participant to be involved in the activity). Other interactions were coded as positive, neutral or restraint. Parent/carer interaction categories were combined to form a single category, Adult Attention. Proximity of another person was defined as Alone (no-one else in the room), Not Close (another person in the room, but at least two metres away) or Close (another person in the room within two metres).

**Table 3:** Behavioural categories and operational definitions

Participant behaviour	
Engaged Activity	
Involving the use of hands	Use of computer, switches, reaching for objects, manipulating toys or objects, taking objects to mouth, educational tasks, leisure, feeding, eating, self-help activity (for feeding, eating, self-help the person must be involved actively in the activity)
Not involving the use of hands	Listening to music, watching a DVD etc.
Social engagement	
Eye contact	Looking at person for at least 3–5 s or more to attract, maintain or end interaction.
Vocalization	Any sound or word to attract, maintain or end interaction
Movements	Defined and clear movements to attract, maintain or end interaction
Disengaged	
Disengaged	Passive or seemingly trivial movements, neither part of a constructive activity nor repetitive enough to constitute stereotypy nor sufficiently intense to constitute self-injury or aggression. Behaviour not directed towards any person or task.
RTT behaviours/mood	
Hand stereotypies	Repetitive movements of the hands that include wringing, tapping, rubbing washing movements, hand mouthing. The movements may be performed with hands together or hands apart.
Other stereotypies	Includes any other repetitive movements such as body rocking, bruxism, repetitive movements with the head, repetitive tongue movements, facial grimacing and repetitive vocalisations.
Self-injurious behaviours	Any behaviour that leads to physical harm or potential harm, including hitting own body, tapping/rubbing own body sufficiently to discolour skin, biting own body, scratching own body, hand biting, hair pulling, skin picking, banging own body (e.g., head) on fixtures (e.g., wall, table).
Aggression	Any physical act towards another person that leads to physical harm or potential harm, includes behaviours such as hair pulling, hitting, breaking property or objects. Any vocal aggression, including screaming, shouting, swearing at another person.
Mobility Mood	Any behaviour when the child is moving around. Clear emotional states: • Positive vocalization/facial expression: i.e. smiling, laughing • Negative vocalization/facial expression: i.e. crying, screaming, sad expression.
Breathing abnormalities Rett Episodes	Hyperventilation, breath hold, valsalva Identified as possible seizure, eye glaze is not fixed, appear not to be breathing, no hand movements, absence of motor activities (non epileptic behaviour)
Giving assistance (Support)	Parent or carer helps the person to do an activity by, verbal or physical prompting, giving an instructing, demonstrating or miming the activity or handing the person objects involved in the activity/placing objects in front of the person or engaged in parallel play/activity (i.e. doing an activity alongside the person as an activity partner). May involve helping the person to feed or drink but, in general, does not involve doing an activity for the person (e.g., brushing hair, washing hands)
Help	Doing an activity to/for the person that involves attention/contact such as feeding, dressing, washing, grooming the person in a way that does not encourage the person’s involvement (i.e. his or her role is passive).
Positive interaction	Parent or carer is interacting with the person in a positive manner but not in a way that gives assistance. i.e. praise, kissing/stroking the person, reading or singing to the person. The parent/carer must be involved with the person, giving attention.
Neutral Interaction	Talking to the person in a way that neither encourages not discourages activity (e.g., greeting the person, incidental remarks, commenting). Or physically contacting the person in a way that neither encourages not discourages activity (e.g., holding hands, having the person sitting on lap).
Restrain**t**	• Prevention: Physical actions or vocalisations to discourage activity (e.g., physical prevention of movements, hold hand to stop stereotypies, telling the person not to do something). • Mechanical restraint: for example the person is wearing an arm splint
Environmental condition	
Alone	Nobody in the room
Not Close (person in the room)	Any person, family member or carer in the room but not close. Defined as being not within 2 m.
Close (person in the room)	Any person, family member or carer in the room and close. Defined as being within 1–2 m.

The observational data were captured on video using a digital camcorder in the participants’ homes and, where relevant, schools or day placements. Arising from practical constraints, the number and length of recording sessions varied between participants. Total times ranged from 1 h and 28 min to 5 h and 30 min, with an overall total across the 10 participants of 29 h and 8 min. The aim was to record the participants’ usual activities (e.g., leisure, meal time, group and individual activities). The person undertaking the recording tried to be as discrete as possible during the sessions so as not to intrude on the activities of the participants. Parents and carers/teachers were instructed to interact with the person as normal. On some occasions, observation had to be stopped because the participant had a seizure, was not well or the presence of an extra person in the environment was too disruptive.

All videos were coded using OBSWIN software (Martin et al. 2001). Observation categories were allocated a key on the computer keyboard, which for convenience was labelled with an abbreviated category name. OBSWIN uses real time analyses in which all categories occur in temporal sequence measured by elapsed time in seconds from the beginning of the session. Times of occurrence correspond to key depressions. The variables under observation can be recorded as events (a single key depression which indicates occurrence during a particular one-second window) or as durations (two key depressions which indicate onset and offset times).

### Inter - Observer Reliability

A second observer coded the first 15 min of each participant’s observation session for inter-rater reliability, giving a total of 2.5 h checked for reliability (8.6 % of the total). In comparing the timing of key depressions (onsets and offsets) between observers, those occurring within a tolerance of 5 s were considered agreements. Cohen’s kappa was calculated for each variable under observation. However, as kappa becomes problematically stringent if a behaviour occurs very rarely or nearly all of the time, percentage occurrence agreement and percentage non occurrence agreement were also calculated.

Table 4 summarises mean Kappa values and occurrence and non occurrence agreement percentages across all variables under observation. Codes were divided into three categories: (a) reliably coded, with a kappa above 0.6, (b) on the margins of being reliably coded, with a kappa between 0.41 and 0.60 and (c) unreliably coded with kappa equal to or below 0.40 (see Landis and Koch (1977) for interpretation of kappa levels). Codes in the last category were excluded from analysis. For codes with a kappa of 0.41–0.60, percentage occurrence and non occurrence agreement figures were examined to determine whether there could be reasonable confidence in the coding. Codes with a kappa in this range without occurrence or non-occurrence percentages above 90 % were excluded from analysis. The aggression, mobility and Rett episodes categories were not observed during the reliability coding and were excluded from analysis.

**Table 4:** Cohen’s Kappa values and occurrence and non-occurrence agreement percentages for the observational categories

	Kappa		% Occurrence	% Non Occurrence
	Mean	Range		
Engaged in activity (hands)	0.74	0.68–1.00	77.2	92.3
Engaged in activities (No hands)	0.93	0.79–1.00	91.3	99.4
Disengaged**	0.59	0.00–1.00	77.3	69.2
Eye contact	0.58	0.00–0.88	50.8	97.1
Vocalization	0.63	0.35–0.92	58.4	99.1
Movements	0.92	0.92–0.92	85.7	100.0
Hand stereotypies	0.78	0.36–0.95	88.1	74.8
Other stereotypies*	0.40	0.12–0.79	44.0	91.7
Self-injurious behaviours	0.48	0.21–0.75	37.5	99.3
Positive Mood*	0.00	NA	0.0	99.1
Negative Mood	1.00	NA	100.0	100
Breathing abnormalities**	0.49	0.00–0.87	62.3	83.7
Giving assistance (Support)	0.82	0.45–1.00	82.3	88.9
Help	0.91	0.70–1.00	90.4	97.1
Positive interactions	0.51	0.00–1.00	50.3	96.8
Neutral Interaction	0.52	0.00–0.89	46.4	94.0
Prevention*	0.35	0.00–0.85	28.0	97.8
Mechanical Restraint*	0.00	NA	98.9	90.9
Alone	0.86	0.76–0.96	80.9	98.3
Not Close	0.71	0.00–0.97	66.0	98.9
Close	0.87	0.74–1.00	98.1	82.4

*Codes excluded due to inadequate kappa

**Codes excluded due to inferior kappa and poor occurrence/non-occurrence reliability

### Data Analysis

Percentage occurrence of each category was calculated by dividing its cumulative duration across bouts (onset times minus offset times) by the total session time. Variability in participants’ levels of engagement in activity or social interaction together with the levels of adult attention each received was analysed in relation to participants’ simplified severity scores and Vineland age equivalent scores using Spearman non-parametric correlation.

Variability of participant behaviour in relation to receipt of adult attention was analysed using lag analysis. The conditional probability of behaviour and attention occurring together was calculated by using a zero lag. In addition, the conditional probabilities of participant behaviour occurring up to 100 s before and after the onset of adult attention were calculated by grouping one second intervals into 10s bins using a partial interval rationale and performing 10 lags in both directions. Conditional and unconditional probabilities were then used to calculate Yule’s Q, a simple arithmetic transformation of the odds ratio (Yoder and Feurer 2000). The significance of Yule’s Q was evaluated with the following equation (Sheskin 2003): z = Q/√[0.25(1-Q^2^)^2^(1/a + 1/b + 1/c + 1/d)] where a, b, c, d are cells of a typical 2 × 2 table. The alpha level for the lag analyses was reduced due to the number of tests performed: a z score above 3.09 (*p* < .001) was considered as a significant level of association.

## Results

### Percentage Occurrence of Behaviour and Environmental Conditions

All participants were observed at home and six were also observed at school or day centre. Three of the five children and the adolescent wore arm splints for some of the time: P1, P2, P3 and P6. In general, participants were mainly in the company of parents, teachers or carers and received attention at a high rate (see Table 5). Parents were in close proximity for about two-thirds of the time at home (median 69.6 %, IQR 34.3 %, range 15.8 % – 90.2 %) and teachers or carers for even longer at school/day centre (median 93.2 %, IQR 11.7 %, range 84.4 % – 99.9 %). Only one participant (P7) spent the majority of the time at home without close proximity (84.1 %). Participants also received adult attention for most of the time at home (median 58.8 %, IQR 46.3 %, range 14.9 % - 86.8 %) and at school (median 78.3 %, IQR 23.5 %, range 56.1 % – 92.4 %). Adult attention came in the form of Support to do an activity for a little over a tenth of the time at home (median 11.7 %, IQR 14.3 %, range 0.1 % - 36.8 %) and for about a third of the time at school (median 32.7 %, IQR 46.0 %, range 0.0 % – 70.0 %). There were no significant differences between child/adolescent and adult participants in these respects. Indeed, comparative median levels for these variables were rather similar.

**Table 5:** Percentage duration of time for each social environmental condition

	Alone		Not Close		Close		Adult Attention	
	Home	School/Centre	Home	School/Centre	Home	School/Centre	Home	School/Centre
Children and Adolescent								
P1	13.1	-	13.1	-	73.7	-	35.5	-
P2	27.5	0.0	4.2	15.3	65.4	84.4	62.2	72.0
P3	0.8	0.0	0.0	9.1	97.5	90.8	82.0	89.4
P4	33.2	0.0	2.9	13.1	63.9	85.8	35.8	70.2
P5	0.3	-	0.7		98.9	-	84.2	-
P6	18.5	0.0	17.3	3.4	64.2	96.2	55.4	92.4
Adults								
P7	63.5	0.0	20.6	3.0	15.8	95.1	14.9	56.1
P8	9.9	-	28.0	-	61.9	-	39.2	-
P9	0.0	0.0	3.8	0.0	96.0	99.9	86.8	84.7
P10	0.0	-	1.4	-	98.4	-	82.0	-

The most common activities in which participants were engaged were: watching television, listening to music, listening to a story book and early learning activities involving simple manipulative toys, switches and water. The percentages of time that participants were engaged in constructive activities at home and at school/day centre are set out in Table 6. Use of the hands at home occupied a median of 12.7 % of the time (IQR 30.0 %, range 0.0 % – 54.1 %) and at school/day centre a median of 17.9 % (IQR 40.8 %, range 2.5 % – 46.6 %). Four participants (P1, P3, P5 and P8) had zero or near zero constructive use of the hands. Engagement in activity involving the use of the hands was inversely related to the simplified severity score both at home and school/day centre (Home: r_s_ = − .64, *p* < .05; School/Day centre: r_s_ = −.93, *p* < .01) and positively related to the Vineland motor skills age equivalent score at school/day centre (r_s_ = .90, *p* < .05).

**Table 6:** Percentage durations of time participants were engaged in activity

	Engaged Activity (hands)		Engaged Activity (no hands)		Social engagement	
	Home	School/Centre	Home	School/Centre	Home	School/Centre
Children and Adolescent						
P1	0.8	-	1.2	-	1.9	-
P2	24.0	21.0	11.6	15.2	22.3	46.0
P3	0.0	4.8	46.7	4.2	10.0	15.1
P4	36.1	14.8	46.2	42.2	20.9	35.5
P5	0.0	-	85.7	-	11.3	-
P6	1.7	2.5	8.5	7.3	1.4	2.8
Adults						
P7	54.1	46.6	13.9	16.8	6.1	13.5
P8	0.0	-	35.8	-	2.7	-
P9	23.6	44.5	57.8	39.2	12.5	7.1
P10	28.0	-	18.7	-	38.7	-

Engagement that did not involve the use of the hands occupied a median of 27.3 % of the time at home (IQR 38.6 %, range 1.2 % – 85.7 %) and 16.0 % of the time at school/day centre (IQR 33.4 %, range 4.2 % – 42.2 %). Such engagement was not related to either the severity score or Vineland equivalent age scores but was related at home to the level of adult attention (r_s_ = .67, *p* < .05) and at school/day centre to the level of support (assistance/instruction) received from teacher or carer (r_s_ = .89, *p* < .05). Engagement in activity either using or not using the hands, (median 46.7 % of the time at home and 46.6 % of the time at school or day centre) was inversely related to the simplified severity score at school/day centre (r_s_ = −.93, *p* < .01), positively related to the Vineland motor skills age equivalent score at school/day centre (r_s_ = .90, *p* < .05) and positively related to receipt of support in both settings (Home: r_s_ = .65, *p* < .05; School/Day centre: r_s_ = .87, *p* < .05).

Social engagement occurred for a median of 10.7 % of the time at home (IQR 18.7 %, range 1.4 % – 38.7 %) and a median of 14.3 % of the time at school/day centre (IQR 32.1 %, range 2.8 % – 46.0 %) and was not related to either the severity score or Vineland equivalent age scores. At home it was positively related to the level of support received (r_s_ = .79, *p* < .01). Only three participants (P1, P6 and P8) were engaged in activity for the minority of the time. Social engagement levels were similar between child/adolescent and adult participants. There were no significant differences in the level of non-social engagement between child/adolescent and adult participants either but there was a tendency for levels of engagement using the hands to be higher among adult participants.

Table 7 summarises the percentages of time participants engaged in hand stereotypies and self-injury at home and in the school/day centre. Hand stereotypies observed included: hand wringing, hand flapping, hand mouthing, hand clapping and holding hands together. All but one participant (P9) were observed to engage in hand stereotypies for appreciable periods, although their extent for a third (P5) was less than for the remaining eight. There was no association between the extent of hand stereotypies and the simplified severity score or developmental age. Six participants were observed to self-injure (P1, P2, P5, P6, P8 and P9). Self-injurious behaviour observed included: biting the hand, biting the arm, biting the fingers, hitting the head with the fist and hitting the mouth. The extent of self-injury was not significantly associated with either the simplified severity score or developmental age.

**Table 7:** Percentage occurrence of hand stereotypies and self-injury

Participants	Hand stereotypies		Self-Injury	
	Home	School/Centre	Home	School/Centre
Children and Adolescent				
P1	62.2	-	6.2	-
P2	71.2	22.9	1.9	1.7
P3	32.5	59.7	0.0	0.0
P4	99.4	89.8	0.0	0.0
P5	19.3	-	8.9	-
P6	61.2	54.1	6.0	1.5
Adults				
P7	33.9	40.9	0.0	0.0
P8	94.6	-	2.8	-
P9	0.0	0.0	0.0	0.9
P10	54.5	-	0	-

### Co-Occurrence of Behaviour and Adult Attention

Six of the 15 Yule’s Q scores between engagement using the hands and adult attention were significantly positive, two significantly negative and seven non-significant (see Table 8). Eight of the 15 Yule’s Q scores between engagement not using the hands and adult attention were significantly positive, three significantly negative and four non-significant. Fourteen of the 16 Yule’s Q scores between social engagement and adult attention were significantly positive and 2 non-significant. Four of the 14 Yule’s Q scores between hand stereotypies and adult attention were significantly positive, five were significantly negative and five non-significant. Five of the seven Yule’s Q scores between self-injury and adult attention were significantly negative, one significantly positive and one non-significant.

**Table 8:** Significant Yule’s Q scores for the co-occurrence between levels of engagement, hand stereotypies and self-injury and receipt of adult attention

Participant	Engaged in activity (Hands)		Engaged in activity (No Hands)		Social Engagement		Hand stereotypies		Self-injury	
	Home	School/Centre	Home	School/Centre	Home	School/Centre	Home	School/Centre	Home	School/Centre
Children and Adolescent										
P1	†	−	*	−	+ 0.91	−	†	−	−0.69	−
P2	+ 0.44	+0.39	†	†	+ 0.87	+ 0.42	−0.70	−0.33	†	−0.61
P3	†	†	†	+ 1.00	+ 0.30	+ 0.56	−0.31	†	−	−
P4	+ 0.51	+ 0.87	- 0.75	+ 0.80	+ 0.80	†	+1.00	−0.48	−	−
P5	†	−	- 0.51	−	+ 0.83	−	+0.93	−	+0.88	−
P6	+ 1.00	+ 1.00	+ 0.32	+ 1.00	+ 0.96	+1.00	−0.50	†	−	−0.92
Adults										
P7	†	- 0.70	+ 0.33	+ 0.77	+ 0.86	+ 0.59	†	+0.69	−	−
P8	−	−	+ 0.52	−	+ 0.63	−	†	−	−0.44	−
P9	†	†	†	- 0.36	+ 0.97	+ 0.42	−	−	−	−0.40
P10	- 0.79	−	+ 0.90	−	†	−	+0.51	−	−	−

^*The two behaviours did not occur together^

^† Non-significant association^

### Sequential Analysis

Time based sequential analysis was conducted to calculate the conditional probability of the presence of the participants’ engagement, hand stereotypies, and self-injury occurring prior to or after the onset of adult attention. There were 18 analyses relating to engagement in activity. In eight, no significant association with adult attention was found and in a further eight the conditional probability of engagement given attention was either consistently or fairly consistently either above or below the unconditional probability of engagement both before and after the onset of adult attention, again suggesting an absence of relationship. In the remaining two analyses, the conditional probability of engagement given attention was significantly reduced prior to receipt of attention and significantly increased afterwards, albeit only temporarily.

**Fig. 1:**
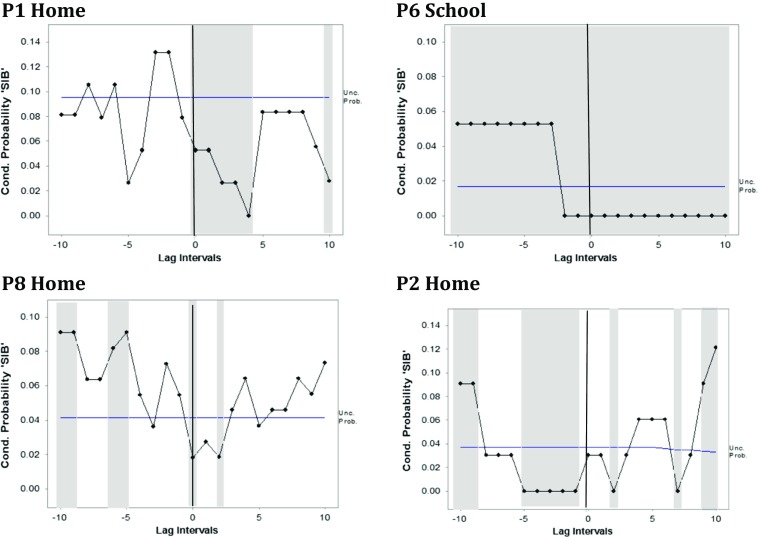
Conditional probability of self-injurious behaviour (SIB) 100 s before and after the onset of adult attention and the unconditional probability of SIB* * The horizontal line indicates the unconditional probability of SIB. The vertical line indicates the onset of adult attention. The dotted line indicates the conditional probability of SIB before and after the onset of adult attention. The shaded area indicates that the unconditional probability is significantly different from the conditional probability (absolute Yule’s Q > 0.3)

The 14 analyses of the relationship between hand stereotypies and adult attention showed either no or no consistent pattern. Self-injurious behaviours were observed in six participants, yielding seven sequential analyses. There was no interpretable pattern in three of these with the conditional probability of self-injury either being significantly below or above the unconditional probability both before and after the onset of adult attention. However, in the remaining four, there appeared to be a relationship between self-injury and receipt of adult attention (see Fig. 1). In three cases (P1 at home, P6 at school and P8 at home), there was evidence of the conditional probability being above the unconditional probability before the onset of attention and below it subsequently, suggesting a possible attention seeking motivation. In the fourth case (P2 at home), there was evidence of the conditional probability being below the unconditional probability before the onset of attention and above it subsequently, suggesting a possible avoidance motivation.

## Discussion

Systematic observation was conducted to explore the frequency of various behaviours manifested by females with RTT and a confirmed *MECP2* mutation. The behavioural observations were conducted in the participants’ everyday environments with a view to analysing the relationship between adult proximity and attention and participants’ behaviour. Although the study is to our knowledge the largest direct observational study of females with RTT undertaken, it has a number of weaknesses. The sample included five children, one of whom had atypical RTT rather than classic RTT, an adolescent and four adults. The age range may be considered a weakness although there was broad similarity in the results between the children/adolescent and adults. The main difference was the suggestion that levels of engagement using the hands was higher among the adults. However, a larger sample is required to compare age groups adequately. Moreover, all participants had a milder severity score and, therefore, generalisation of the findings has to be limited. The extent of data collection per person and the activities represented within each participant’s dataset were not standardised as practical constraints dictated that there was variation between participants. A subsequent study might ensure greater uniformity in these respects and have the capacity to analyse different types of activity period separately. Further development is also required to establish a definitive behavioural coding scheme with sufficiently good inter-observer agreement on all categories of interest. In addition, it is not known what effect filming as a means of data capture may have had on the events being recorded.

The one child with atypical RTT may be considered different to the remaining participants. She was also one of the two youngest participants. She had the highest severity score, which at nine was on the border of the transition from mild to severe, but she was cognitively the most able. She was the only participant to have some speech and she had the highest Vineland composite standard score and second highest developmental age, despite her youth. Like other participants, she spent most of her time in the company of a parent, teacher or carer but received the second lowest level of adult attention. Her level of engagement in constructive social or non-social activities was particularly low (less than 5 % of the time). She spent about two-thirds of the time engaging in hand stereotypies, nearly three-fifths of the time in arm splints and was also one of the participants who self-injured. Her self-injury appeared to be attention-seeking. It occurred significantly less often when she was receiving adult attention and the lag analysis also suggested an attention-seeking motivation.

Compared to data from residential settings for individuals with a similar severity of intellectual disability (e.g., Emerson et al. 1999, 2000), individuals with classic RTT in this study received a high level of adult attention in the form of positive interaction, assistance or help. In addition, their levels of social interaction and engagement in activities were also relatively high. Clearly, one cannot make comparisons between different types of environment without taking account of contextual factors such as the ratio between those needing support and those in support roles. However, the above comparisons do seem to indicate that the classic RTT participants in this sample had a relatively rich social environment. After the regression stage, individuals with RTT are reported to be sociable and to be responsive to their environment. Such characteristics may help sustain a conducive social environment.

Engagement in activity has been much studied in residential settings and there is fairly consistent evidence that there is a primary association with individual ability as measured by an adaptive behaviour scale and a secondary association with care practices, as measured by the extent of practical support individuals receive or the implementation of ‘active support’ (Felce and Perry 1995; Felce and Emerson 2001; Felce et al. 2003; Mansell et al. 2003; Perry and Felce 2005). Findings here were consistent with this evidence to some extent. Engagement in activity using the hands was associated with a less severe condition and greater adaptive behaviour, engagement in activity not using the hands occurred most commonly with receipt of adult attention among the majority of participants, and engagement in activity whether using the hands or not and social engagement were associated with receipt of support. However, a relationship between non-social engagement in activity and receipt of attention was not demonstrated in the sequential lag analyses.

Stereotyped hand movements are considered to be characteristic features of RTT and among the essential diagnostic criteria. Despite this, the duration of hand stereotypies observed varied across the sample, from a very limited extent to almost all of the time. Variation appeared to be independent of social context. The correlation between the extents of hand stereotypies and adult attention was insignificant and there were no consistent associations between hand stereotypies and adult attention revealed in the concurrent or sequential lag analyses. This apparent independence from environmental influence is consistent with the findings of Wales et al. (2004) who demonstrated that the hand stereotypies of eight girls with RTT were not susceptible to environmental manipulation.

Self-injury is not an essential diagnostic criterion for RTT but has been shown to occur in a reasonably high proportion. Self-injury was found to occur more frequently among individuals with a severity score in the mild range among the national sample from which this sample was drawn (Cianfaglione et al. 2015a). This may explain why as many as six (five with classic RTT and one atypical RTT) out of the ten participants in this study were observed to self-injure. As in Oliver at al. (1993) there was evidence that self-injury may be socially motivated.

Despite the inconsistency of results across participants, the study has highlighted the importance of considering the role of the environment in shaping the behaviour of females with RTT. The participants in this study appeared to experience a carer and attention rich environment and their levels of engagement were likely to be higher than in some other studies as a result. As in the more general literature, engagement in activity was related to personal development and to social support. Hand stereotypies are characteristic, although varying considerably across participants, and appear unrelated to environmental events. Self-injury in RTT appears to occur for reasons similar to that in other individuals with intellectual disability. It was found to be associated with overactivity and impulsivity (Cianfaglione et al. 2015a) and may serve attention-seeking or social avoidance purposes.
